# Targeting Adult Neurogenesis for Poststroke Therapy

**DOI:** 10.1155/2017/5868632

**Published:** 2017-07-20

**Authors:** Jianfei Lu, Anatol Manaenko, Qin Hu

**Affiliations:** ^1^Discipline of Neuroscience, Department of Anatomy, Histology and Embryology, Shanghai Jiao Tong University School of Medicine, Shanghai 200025, China; ^2^Department of Anatomy and Embryology, School of Basic Medical Sciences, Peking University Health Science Center, Beijing 100191, China; ^3^Departments of Neurology, University of Erlangen-Nuremberg, Erlangen, Germany

## Abstract

Adult neurogenesis mainly occurs at the subventricular zone (SVZ) on the walls of the lateral ventricle and the subgranular zone (SGZ) of the dentate gyrus (DG). However, the majority of newborn neurons undergo programmed cell death (PCD) during the period of proliferation, migration, and integration. Stroke activates neural stem cells (NSCs) in both SVZ and SGZ. This process is regulated by a wide variety of signaling pathways. However, the newborn neurons derived from adult neurogenesis are insufficient for tissue repair and function recovery. Thus, enhancing the endogenous neurogenesis driven by ischemia and promoting the survival of newborn neurons can be promising therapeutic interventions for stroke. Here, we present an overview of the process of adult neurogenesis and the potential of stroke-induced neurogenesis on brain repair.

## 1. Introduction

Stroke is one of the leading causes of morbidity and mortality worldwide. In addition, about two-thirds patients had neurologic impairment and disability, based on a population study of follow-up of stroke survivors at five years [1]. Therefore, poststroke rehabilitation becomes a major therapeutic focus for most poststroke patients. Unfortunately, the currently available therapies are only rarely successful in improving recovery from neurological deficits. It is well established that de novo neurogenesis mainly occurs at two distinct regions in the adult brain: the SGZ of the dentate gyrus of the hippocampus and the SVZ adjacent to the lateral ventricle [2, 3]. In pathological conditions such as stroke, increased neurogenesis has been reported in adult animal models and even in stroke patients [4]. The proliferated neural progenitor cells migrate to the injured striatum and cortex; however, most of them failed to survive and rewire the brain. Taking advantage of the neurogenic capacity of the brain and improving the survival of endogenous neuroprogenitor cells shed light on the restorative therapies for stroke and other brain insults. Here, we review adult neurogenesis from a comprehensive perspective and summarize the current status of research on neurogenesis in poststroke therapy.

## 2. Adult Neurogenesis

Adult neurogenesis (AN) is a process that is continuously producing new neurons which integrate into existing circuits in adult age and have different mechanisms compared with fetal and early postnatal development [5]. AN was first demonstrated by Altman and Das in a rat brain in 1965 [6]. They injected thymidine-H^3^ into adult rats and cats to tag the newborn cells and found that the labeled glia cells and neurons are present in various regions of the normal adult mammalian brain. In the 1990s, bromodeoxyuridine (BrdU) was applied to label newborn cells in neurogenesis research. With the application of this new technique, two areas of the adult neurogenesis were found: the DG and the SVZ. In the DG, new neurons continue to be generated from NSCs in the SGZ. NSCs also reside and proliferate in the SVZ and differentiate into neuroblasts. These neuroblasts migrate through the rostral migratory stream (RMS) to the olfactory bulb (OB) and integrate into OB circuits. Recently, some noncanonical sites of adult neurogenesis, such as neocortex, striatum, corpus callosum, amygdala, and hypothalamus, have been found in different species [7].

The process of maturation of new neurons encompasses the proliferation of resident NSCs and their subsequent differentiation, migration, survival, and functional integration into the preexisting circuitry [8]. AN is mediated by a series of physiological and pathological processes at all these stages. Moreover, programmed cell death (PCD) plays critical roles in regulating the process from NSC proliferation to the integration of neural circuits. We focus on current knowledge of the main neurogenic sites (SVZ and SGZ) of AN with their specificities and address the potential roles of PCD as a regulatory strategy.

### 2.1. AN in SVZ and SGZ

#### 2.1.1. SVZ

**Figure 1:**
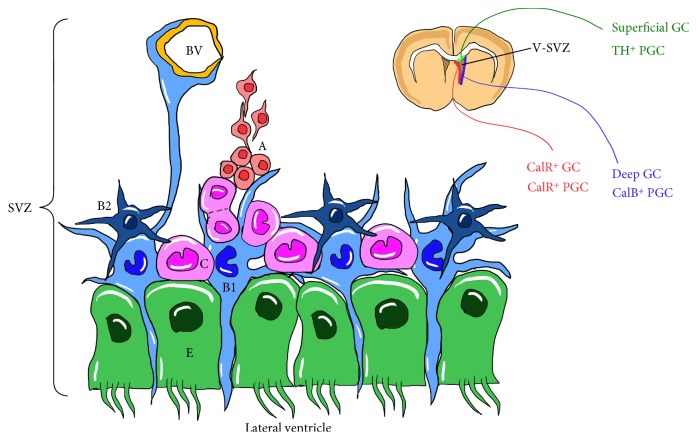
Neurogenesis in SVZ. The SVZ is shown in the left. Type B1 cells (B1, blue) lie atop ependymal cells (E, green) and extend their processes to blood capillary (BV). Type B1 cells divide to produce type C cells (C, pink). Type C cells then give rise to type A cells (A, red). Type B2 cells (B2, dark blue) also reside in the SVZ. The coronal section in the upper right is shown the diversity of newborn OB interneurons. Deep GCs and CalB^+^ PGCs are derived from ventral NSCs, whereas superficial GCs and TH^+^ PGCs are derived dorsal NSCs. NSCs from the medial wall produce CalR^+^ GCs and CalR^+^ PGCs.

In mammalian animals, new OB neurons are derived from SVZ, on the walls of the lateral ventricles. The SVZ have five main cell types: B1 astrocytes (type B1 cells), B2 astrocytes (type B2 cells), transit-amplifying cells (type C cells), neuroblasts (type A cells), and ependymal cells (type E cells) (Figure 1). Microglia and oligodendrocyte precursor cells (OPCs) also reside in the SVZ. Type B2 cells and ependymal cells are important for maintaining and regulating the niche of SVZ. Type B1 cells lie atop ependymal cells and extend their processes further to blood capillary [9]. Besides, most B1 astrocytes contact the ventricle by extending a thin cellular process between ependymal cells [2]. Type C cells are shaped like a smooth ellipse and have large nuclei with deep invaginations [2]. Type A cells have an elongated cell body with smooth contours. They have one or two processes and join to other type A cells by small junctional complexes. Nestin, SRY-box 2 (Sox2), and brain lipid-binding protein (BLBP) have been considered as NSC markers. Distal-less homeobox 2 (DLX2), epidermal growth factor receptor (EGFR), and mammalian achaete-scute homolog 1 (MASH1) are mainly expressed on type C cells [10]. Doublecortin (DCX), *β*-III-tubulin (TuJ1), and polysialylated neural cell adhesion molecule (PSA-NCAM) are the unique markers of type A cells [2].

NSCs in the SVZ correspond to type B1 cells. Asymmetric division of type B1 cells produce self-renewed type B1 cells and type C cells [11, 12], which symmetrically divide into type A cells [13]. After birth in SVZ, type A cells form elongated, chain-like aggregates, which are ensheathed by astrocytes [14–16]. These neuroblasts migrate through RMS at the anterior SVZ [17]. The migration of neuroblasts follows a salutatory manner: first, a leading process extended; then, swelling formation and centrosome migration; and last, somal translocation [16, 18–20]. The RMS carries the neuroblasts into the OB where these neuroblasts detach from the RMS and then migrate radially to the outer layer and differentiate into various subtypes of olfactory neurons.

There are two principal types of adult-born OB neurons: periglomerular cells (PGCs) in the glomerular layer (GL) and granule cells (GCs) in the granule cell layer (GCL). Deep GCs and calbindin (CalB)^+^ PGCs are derived from ventral NSCs, whereas superficial GCs and tyrosine hydroxylase (TH)^+^ PGCs are derived from dorsal NSCs. NSCs from medial wall produce calretinin (CalR)^+^ GCs and CalR^+^ PGCs [21] (Figure 1).

#### 2.1.2. SGZ

**Figure 2:**
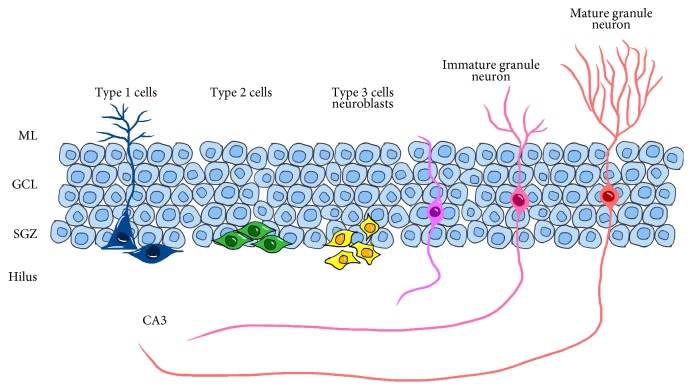
Neurogenesis in SGZ. The SGZ is a thin band of tissue that lies between the granule cell layer (GCL) and the hilus cells in the DG. Type 1 cells are triangular-shaped NSCs and usually extend a strong apical process into the molecular layer (ML). Type 1 cells (blue) generate type 2 cells (green). Type 2 cells are immature neuroblasts that can be further differentiating into type 3 cells (yellow). Type 3 cells progressively acquire characteristics of granule neurons. During the stage of immature (pink) to mature (red), large parts of the dendritic tree and axon elongate toward CA3.

The adult neurogenic niche of the hippocampus resides in the SGZ, a thin band of cells lying between the hilus cells and the granule cell layer in the DG. NSCs first develop into radical astrocytes (type 1 cells) that, in turn, generate intermediate neural progenitors (type 2 cells). These cells are immature neuroblasts that can be further differentiated into neuroblasts. Neuroblasts can be further divided into more differentiated cells (type 3 cells) [22, 23]. Type 3 cells progressively acquire characteristics of neurons. During the stage of immature to mature, elaborate dendritic arborization grows to the middle of the molecular layer and axon elongate toward CA3 [23] (Figure 2).

Type 1 cells are located in the SGZ and have a triangular-shaped soma. A strong apical process extended into the molecular layer of DG is the typical characteristic of type 1 cells. Type 1 cells have some astrocyte features that may contact blood vessels through the end-feet [22]. Recently, another class of type 1 cells has been identified. These newly identified type 1 cells are characterized by short, horizontal processes [24]. Type 2 cells have a unique morphology that is distinct from type 1 cells: they lack the strong apical process and have a round or ovoid nucleus. Type 3 cells have variable morphologies. The processes of type 3 cells are short and the orientations alter from horizontal to vertical (Figure 2). Type 1 cells express GFAP, nestin [25, 26], BLBP, and Sox2. Type 2 cells have both neural and glial features that express neuronal (DCX and PSA-NCAM) and glial marker (nestin, BLBP) [27]. DCX, PSA-NCAM, NeuroD, and Prox1 are mainly expressed on type 3 cells.

### 2.2. PCD for the Regulation of Adult Neurogenesis

PCD is the death of a cell in any form, mediated by an intracellular program that mainly occurs during embryo/adult development and in some pathologic conditions [28]. The majority of adult-born neurons are eliminated by apoptosis. There are three main functions of PCD during adult neurogenesis: (1) regulate of the size of the NSC pool, (2) correct the errors during proliferation and migration, and (3) form correct synaptic contacts. The roles of all three functions are to optimize the neural system.

Neuroblasts from SVZ migrate through RMS to the OB and differentiate into GCs and PGCs. GCs are mature at 15–30 days and PGCs at 4 weeks after birth. There are around 30,000 newborn interneurons integrated into OB neural circuits daily in adult mice [10, 14, 29, 30]. However, 50% of NSCs, neuroblasts, and newborn interneurons undergo apoptosis to eliminate redundant and false connected cells. The survivals integrate into neural circuits and persist up to 19 months [30, 31]. Hippocampal NSCs proliferate and differentiate into granule neurons in the DG. In addition, about 30–70% of the newborn cells die of PCD in the first 2 weeks after birth. The remaining forms functional synapses on CA3 pyramidal neurons at 2 weeks after birth, and this projection becomes stable at 4 weeks [32]. About 4 weeks after birth, dendritic processes of newborn neurons extend toward and into the molecular layer and an axon project into the hilar area [33]. At 2 months, the number of DG neurons in *bax^−/−^* mice has no difference compared with that in wild-type (WT) mice, whereas at 12 months, the number of DG neurons is doubled in *bax^−/−^* mice [34]. In adult humans, 700 new neurons are added to the hippocampus per day and with a continuous decline during aging [35]. These data indicate that PCD is important for the renewal of neural circuits and occurs at all stages during adult neurogenesis.

#### 2.2.1. PCD of NSCs

Growth factors secreted in the SVZ niche are essential for the survival of NSCs. Thus, NSCs that lack neurotrophic signals are more sensitive to apoptosis stimuli. NSCs from adult *bax^−/−^bak^−/−^* mice show resistance to a series of apoptotic stimuli and are accumulated in the SVZ and SGZ. *Bax* single-deficient NSCs are resistant to apoptosis induced by staurosporine [36]. While in *Mcl1* conditional knockout mice, NSCs in the SVZ are more vulnerable to apoptotic cell death. However, overexpression of Mcl-1 reduces the apoptotic rate by about 50% in NSCs from the SVZ [37]. Regarding all above results, Bcl-2 family proteins play an essential role for the apoptosis of NSCs and regulate the size of the NSC pool. In the DG, *bim* or *puma* deficiency significantly enhanced the survival of adult-born cells but have no change on NSC differentiation [38]. *Puma* deficiency also increases the survival of SVZ NSCs. Puma is required for p53-induced apoptosis in NSCs of DG [39, 40]. Besides, loss of *Trp53* enhances slow and fast proliferation in SVZ populations and associates with their differentiation toward neuronal and glial cell lines [41]. However, opposite results are found in the mice knockout *Trp53* and p53 deficiency induces apoptotic brain lesion. These *p53*-deficient mice have thinner isocotex and enlarged ventricle compared with wild-type mice [42]. Therefore, the exact role and mechanism of p53 in regulating the PCD of NSCs remain unclear. Adult hippocampal NSCs undergo autophagic cell death instead of apoptosis on deprivation of insulin [43–45].

#### 2.2.2. PCD during Migration and Integration

Errors during migration also induce apoptosis in the adult-born neuroblasts. In *bax*-deficient mice, a large number of abnormal neuroblasts accumulate in the RMS [10]. A similar result exists in newborn cells with increased mTOR activity. Heterotopia and ectopic neuroblasts are observed in the RMS and the OB. Moreover, these heterotopia cells survive and integrate to the OB network. They have increased dendritic complexity, altered membrane biophysics, and increased frequency of GABAergic synaptic inputs [46]. However, the effects and functions of these heterotopia and ectopic survived interneurons are still unknown.

The most extensive apoptosis of newborn neurons occurs during the integration into neural circuits. 30–70% of immature neurons are eliminated by apoptosis during the formation of synaptic contacts [29, 31, 47]. This phenomenon can be interpreted by a neurotrophic hypothesis that the neurotrophic substance released for the survival of neurons is limited; thus, newborn neurons need to compete for these trophic signals [48, 49]. Competition for neurotrophic signals not only occurs between homogeneous neuroblasts and immature neurons but also is observed between immature neuron and preexisted mature neuron for new synaptic connections. Using fluorescent retrograde tracers and BrdU-labeling techniques, it is proved that newborn neurons in the DG extend axons into CA3 of hippocampus and may influence the normal hippocampal function [23, 31, 50, 51]. In *bax^−/−^* mice, apoptosis is inhibited in immature and mature neurons of DG, and the size of DG neurons enlarges continuously with age [34]. Synaptic connections with efferent and afferent neurons are both observed in this the DG [10]. All these results proved that the immature neurons can extend axons to mature neurons and make contacts. Thus, the apoptosis of adult-born cells is to keep the balance of mature and immature neurons and maintain the integrity of neuronal circuits [52]. However, some opposite data have shown that in *bax*-deficient mice, the pattern separation function of the hippocampus is enhanced. However, knockout *bax* seems to have no effects on other major hippocampal functions [53]. It seems that pattern separation is regulated by immature DG neurons. Other hippocampal functions, such as aligning internal spatial representation to external landmarks, are mediated by mature DG neurons [54]. A similar phenomenon has also been found in the cell replacement of OB. In *bax^−/−^* mice, the normal olfactory learning behavior is improved, and the perturbations of newborn cell migration result in imbalance of neural circuits that destroy the olfactory learning ability. Besides, the *bax*-deficient mice show no significant changes on olfactory sensation [55, 56]. Based on above results, we may conclude that the immature neurons and mature neurons have different roles in neural circuits, and apoptosis is the key regulator that keeps the balance of adult-born neurons and the preexisting ones.

#### 2.2.3. PCD of Mature Neurons

Although the majority of cell types that undergo PCD are immature neurons and neuroblasts, mature neurons also have lower levels of PCD in the OB and DG. The purpose of PCD in mature neurons is to renew the preexisting neural circuits [30, 57]. At about 15–30 days after birth, newborn cells differentiate into mature neurons in the OB. Thereafter, about 50% newborn neurons undergo apoptosis. Cells that survive the first 3 months persist up to 19 months [31]. One fourth of the DG neurons born at the peak of DG development on postnatal day 6 died in the first 1 to 6 months [58]. The production of adult-born neurons and elimination of mature neurons are critical for the maintenance of a constant number of neurons in DG and for the regulation of hippocampal functions [34].

## 3. AN and Stroke Recovery

Adult NSCs in neurogenic regions can be activated by different stimuli such as learning [59] and running [60] and also can be activated in the disease processes including seizure [61], mechanical lesions [62], and ischemic insult [63]. These results raised the possibility that functional deficits induced by stroke may be cured through neuronal replacement by endogenous NSCs. In well-studied rodent models of stroke, cerebral ischemia and hemorrhage have been shown to stimulate proliferation of endogenous progenitor cells and differentiate into neural system cells, including neurons, astrocytes, oligodendrocytes, and ependymal cells [64]. Evidence for stroke-activated neurogenesis has also been reported in the stroke patients [65]. Accumulating evidence has convincingly demonstrated that stroke-induced neurogenesis in SVZ and SGZ and other noncanonical stem cell niches have also been confirmed in the adult brain.

### 3.1. Classical Neurogenic Niches after Stroke: SGZ and SVZ

In models of transient global cerebral ischemia, cerebral blood flow is reduced throughout the whole brain [66]. The hippocampus CA1 area plays an important role in cognitive processes such as learning and memory and is more sensitive to hypoxia-ischemia insults than other areas of the brain [67, 68]. Remarkable increased progenitor proliferation in hippocampal SGZ has been observed in many species, such as mice, rats, gerbils, and monkeys, after global cerebral ischemia [69–71]. Liu et al. first reported increased hippocampal neurogenesis after transient global ischemia in gerbils in 1998 [63]. Newborn cells with neuronal features were first seen 26 days after ischemia, migrated from the SVZ to the granule cell layer, and survived for at least 7 months [63]. Since this initial publication, many follow-up studies have confirmed stimulation of neurogenesis in the SGZ across various species of global ischemia [69–71]. Nakatomi et al. further revealed that ischemia-induced adult neural progenitors in DG can replace CA1 pyramidal neurons form functional synapses and integrated into the existing brain circuitry [72]. Tanaka and his colleagues visualized that the neuronal progenitor cells in the DG proliferated, migrated, and differentiated into mature neurons by retroviral vector expressing enhanced green fluorescent protein (EGFP) [73]. Increased NSC proliferation has also been reported in the SVZ following global ischemia [74]. Promoting endogenous neurogenesis in SGZ may contribute to replace the CA1 neuron loss and improve function recovery after global ischemia. However, it is also worth to point that some studies cannot reproduce the evidence that SGZ neural stem cell migrate into CA1 as previously reported by Nakatomi and coworkers. In contrast, the CA1 area merely displays gliogenesis [71, 75].

In focal brain ischemia, middle cerebral artery occlusion (MCAO) is the most frequently used focal brain ischemia model, which produces consistent infarcts in the ipsilateral hemisphere of the cerebral cortex, hippocampus, and striatum [76]. Neurogenesis was increased bilaterally in both SVZ and SGZ after unilateral MCAO, indicating that endogenous neuronal precursors might be in response to contralateral ischemia as well [77]. The vast majority of adult neurogenesis in mammalian species occurs within the SVZ. SVZ are a paired brain structure situated throughout the lateral walls of the lateral ventricles. Significant enhanced proliferations of NSCs in the SVZ were observed in the first 7–14 days after MCAO in mice [78] and rats [77, 79, 80]. A fluorescent tracing of proliferating cells in the SVZ showed that these cells directly migrate from birth site to striatum in the post-MCAO rat brain [81]. In the normal brain, most of the SVZ neuroblasts migrate through the RMS into the OB and differentiate into interneurons. Ischemia may revoke the normal migratory pattern of SVZ NSCs and lead these cells to migrate toward the injured areas and aid in spontaneous recovery [82]. In the damaged striatum, neuroblasts were continuously generated from SVZ precursors as early as 1 week and last to 16 weeks after insult [82]. The SVZ was the principal source of the neuroblasts migrated laterally toward the injured striatal regions and integrated into neuronal networks receiving synaptic input and firing action potentials after MCAO [83, 84]. Inspiringly, Kreuzberg and his colleagues found MCAO also induced SVZ-derived neuroblasts migrated to the cortex, differentiated into mature neurons, and survived for at least 35 days [78]. These results highlight the role of the SVZ NSCs in neuronal regeneration after focal cerebral ischemia and its potential as a new therapeutic target for various neurological disorders.

### 3.2. Adult Neurogenesis from Noncanonical Sites

Above studies have shown convincing evidence of neuroblasts migrating from the SVZ or SGZ to the ischemic areas. However, several studies have proposed the possibility that there exist other stem cell niches in the adult brain [85, 86]. In fact, this noncanonical site of adult NSCs has been found in rodent striatum [87], hypothalamus [88], neocortex [87], amygdala [87], substantia nigra [89], and brainstem [90]. In addition, endogenous brain repair occurs in, but not restricted to, neurogenic regions. It is reported that astrocytes surrounding the infarct core lesion can be activated to generate neurons [91, 92]. Pericytes and OPCs have also been reported to differentiate into neurons following brain injury [93]. These data indicate that stem cell niches are much extensive. These existed multiple neurogenesis sites may be important for brain repair after injury [94].

## 4. Targeting AN as Therapeutic Strategy for Stroke

AN has arisen great interest as it can be applied for new therapies to replace damaged neurons and treat severe neurological deficits after stroke and other neurological diseases. However, the endogenous neurogenesis failed in producing adequate amounts of newborn neurons that can survive and integrate to restore the function recovery. Stimulating or enhancing the endogenous neurogenesis driven by ischemia can be a promising therapeutic intervention for stroke. The vast majority of the newborn neurons die between 2 and 5 weeks, which may be caused by the unfavorable environment that is exposed to the detrimental injury niche, lacking appropriate trophic support and failed connections with other neurons after stroke [95]. Reducing endogenous toxic substances, inhibiting inflammatory responses, and promoting the release of growth factors and neurotrophic signals have been suggested as effective manipulations to improve AN.

A wide variety of signaling pathways are related to NSC activity during proliferation, migration, differentiation, and their maintenance in the adult neurogenic regions. As survival is the first factor of newborn neurons, neuroprotective agents or manipulations attempt to benefit from neuronal survival preserving the property to support neurogenesis in long term [96]. Administration of EPO (5 U/g) significantly preserves hemispheric brain volume 6 weeks after stroke and directs cell fate toward neurogenesis and away from gliogenesis [97]. The PI3 kinase/Akt pathway showed to play an important role in neuronal survival as well as adult neurogenesis. Mutation that produces constitutive activation of the Akt pathway through PTEN deletion induces a robust increase in poststroke neurogenesis [98]. Studies that targeted NOTCH, WNT, and sonic hedgehog (SHH) signaling showed the great potential of these approaches in stroke treatment [99–102]. In addition, many growth factors have been identified to protect NSCs and enhance neurogenesis after stroke. Basic fibroblast growth factor (bFGF), epidermal growth factor (EGF), BDNF, bone morphogenetic protein (BMP), glial cell-derived neurotrophic factor (GDNF), transforming growth factor- (TGF-) *α*, ciliary neurotrophic factor (CNTF), and platelet-derived growth factor (PDGF) have all been proposed to play essential roles in the adult neurogenesis response to ischemia stroke [103–111]. Recently, some cytokines and hormones such as chemokine, complement, estrogen, and granulocyte-colony stimulating factor (G-CSF) have been proved to benefit against a stroke-induced brain and behavioral pathology [112–116]. The chemokine stromal-derived factor 1 (SDF1) is induced in peri-infarct blood vessels and serves as a tropic signal for migrating neuroblasts to localize to the ischemic area. Administration of SDF1 improves poststroke neuroblast migration and behavioral recovery [114]. Though great progress has been made, the search for strategies and pharmacological agents to enhance endogenous neurogenesis despite a detrimental milieu remains a challenge and is the focus of intense investigation.

## 5. Concluding Remarks

Majority of stroke patients suffer from serious morbidity and never regain full functional independence. The limited result of stroke treatment has driven the search for stem cell therapies directed at restoring neurological function. However, both technical and ethical issues limit the development of exogenous stem cell therapy [4]. The finding of endogenous neural stem cells in the mammalian brain is a breakthrough and provides a promising approach to repair the damaged lesion after stroke. Great efforts have been made to augment the innate neurogenic capacity of the adult brain, including increasing the survival of NSCs in the neurogenic regions, strengthening their mobilization, and integrating into damaged neural circuits. However, there are issues raised about the AN after stroke. First, little is known regarding the intrinsic properties and the modulation of the NSC fate. Additional research is needed to identify the NSC fate determinants, which modulate the differentiation of NSCs toward specific cell types. Second, the integration of newborn neurons into preexisting neural circuits and the related functional recovery should be studied and improved in future researches [117]. Third, most animal studies of stroke are performed in young adult animals; however, human stroke most frequently occurs in aged patients. Neurogenesis both in the SVZ and SGZ drops precipitously with age, and the effects of age on AN should be considered. Forth, for efficient repair, it may be necessary to provide endogenous and/or graft new cells to form synthetic extracellular matrix so that they can reform appropriate brain structure [118]. The discoveries reported in this review may pave the way for targeting AN as future therapeutic interventions for stroke as well as other central nervous system diseases.
